# The effects of three environmental factors on building evacuation time

**DOI:** 10.1016/j.heliyon.2024.e27128

**Published:** 2024-02-24

**Authors:** Erica Kinkel, C. Natalie van der Wal, Serge P. Hoogendoorn

**Affiliations:** a Department Transport & Planning, Faculty of Civil Engineering and Geosciences, Delft University of Technology, Stevinweg 1, 2628 CN, Delft, the Netherlands; b Department Multi-Actor Systems, Faculty of Technology, Policy and Management, Delft University of Technology, Jaffalaan 5, 2628 BX, Delft, the Netherlands

**Keywords:** Evacuation, Building evacuation time, Fire alarm, Visibility, Emergency exit signage, Experiment

## Abstract

Building fires can be considered a risk to the health and safety of occupants. Environmental factors in building fires might affect the speed of an evacuation. Therefore, in this study participants (*N* = 153) were tested in an experimental design for the effects of (1) a fire alarm, (2) darkness and (3) the use of emergency exit signs on building evacuation time. In addition, the effects of age and gender on evacuation time were investigated. The main results indicate that the combination of a fire alarm, darkness and not illuminated emergency exit signs had a significant negative influence on evacuation time, namely an increase in evacuation time of 26.6% respectively 28.1%. Another important finding is that age had a significant negative effect on evacuation time. The increase in evacuation time was at least 30.4% for participants aged 56 years or older compared to participants aged 18–25 years. For gender no significant effect was found. Building and safety managers can use these results by including longer evacuation time considerations – based on darkness and older age – in their evacuation plans. Future research should focus further on investigating the effects of personal and psychological characteristics on evacuation behaviour and evacuation time.

## 1 Introduction

Building fires can be considered a risk to the health and safety of occupants. Recent fire incidents show that people still perish during building fires. For instance, a fire in a former warehouse in Oakland, California, claimed 36 lives [1], in the Grenfell Tower in London 72 people perished [2] and in a shopping mall and entertainment complex in Kemerovo, Russia, the official death toll was 60 people [3]. In fire situations, environmental factors such as (1) ignoring fire alarms [4,5], (2) reduced or limited visibility in corridors [[6], [7], [8]] or (3) low visibility or ineffective use of emergency exit signs [7,[9], [10], [11]] might affect the speed of an evacuation. These factors have all been studied before separately but, to the knowledge of the authors, not in combination in one experimental setup. Also, there seems to be a lack in quantifiable data about the effect of these environmental factors (combined) on evacuation time. Therefore, in this paper the effects of these three environmental factors on building evacuation time are investigated in an experimental design.

Fire alarms may be ignored because of failure to recognize the signal as a fire alarm or to hear the signal, but occupants could also experience alarm fatigue due to frequent fire drills or previous false alarms [4,12]. For example, the latter was the case in an apartment building fire in New York City. Here, residents heard the fire alarm but did not immediately react because they had grown accustomed to frequent alarms in the building [5]. Also, occupants might underestimate how fast a fire can spread [[13], [14], [15]] or how toxic smoke can be. Low oxygen levels, for instance, can cause headache, dizziness, nausea and/or fatigue and people might become incapacitated and/or unconscious and eventually, at very low levels, it is possible that they perish [16,17]. The latter was the case in the Grenfell Tower where most of the victims died from carbon monoxide inhalation [8]. This shows how dangerous underestimation of a fire can be. Therefore, time is of the essence in a fire emergency evacuation and it is important that occupants immediately evacuate the building when a fire alarm is activated. However, previous studies of real incidents suggest that fire alarms do not urge occupants to take immediate action and evacuate the building [[18], [19], [20]]. Although fire alarms are used in experimental studies [21,22], the effect on building evacuation time is rarely investigated.

Reduced or limited visibility due to smoke and/or darkness could make wayfinding more complex or might even slow down the walking speed during evacuation [7,23,24]. In smoke-filled environments this seems to be the case with a lighting level below 3 lux [25,26]. Also the combination of smoke and not illuminated corridors seems to slow down walking speed significantly [27]. On the other hand, in dark but smoke-free environments the amount of lighting also might affect walking speed during evacuation. According to Boyce [28] the walking speed starts to be influenced at lighting levels below 1 lux. In addition, age can have a negative effect on walking speed in dark and smoke-free environments [25,26,28]. Older evacuees appear to be slower than younger evacuees. Furthermore, some studies suggest that gender can also have an effect on reaction times and walking speed during evacuations [29,30]. Most of these studies are focussed on walking speed. What the effect of reduced or limited visibility due to darkness is on building evacuation time is, however, still unclear.

Smoke may also prevent the visibility and use of emergency exit signs [9,10,[31], [32], [33]]. Some experiments demonstrated that the effect of emergency exit signs is significantly stronger when no smoke is perceptible than in smoke-filled environments [9] and that the average signage detection probability under normal conditions is larger than under evacuation conditions [34]. But in real fires, only a few people (between 6 and 8%) notice exit signs during emergency egress [35]. In evacuation experiments in normal conditions there seems to be a difference in the detection of *static* emergency exit signs compared to *dynamic* emergency exit signs. Xie et al. [36] performed an evacuation experiment and found that only 38% of the participants unfamiliar with the building layout detected the static emergency exit signs. Galea et al. [37] tested the use of a dynamic signage system in the same experimental environment as Xie et al. [36] but now they used an emergency exit sign with a flashing arrow. The results show that the dynamic system had a detection rate of 77% when the participants directly approached the sign. Similar results were found in follow-up experiments with an active dynamic signage system [[38], [39], [40]]. Also, Meng and Zhang [41] found that it took stressed participants longer to detect evacuation signs and they had a longer evacuation time compared to non-stressed participants. Furthermore, there seems to be a difference in the signage detection rate between individuals and groups [34]. The detection and compliance probabilities of emergency signs under individual conditions seems larger than those under group situations, probably because of social influence in groups [34]. Nevertheless, there are still knowledge gaps about the effect of emergency exit signs on building evacuation time.

To address some of the limitations mentioned in the above discussed literature, this study uses an experimental design in which three different evacuation scenarios are simulated and compared with a neutral evacuation scenario to determine the effects of three environmental factors on evacuation time. The experimental evacuation conditions are: (1) the use of a fire alarm in normal lighting conditions and with illuminated emergency exit signs, (2) the use of a fire alarm combined with a dark environment and illuminated emergency exit signs and (3) the use of a fire alarm combined with a dark environment and not illuminated emergency exit signs. In addition, the effects of personal characteristics (age and gender) on evacuation time will be analysed. The research questions are.

1. What are the effects of three environmental factors, namely a fire alarm, darkness and not illuminated emergency exit signs, on building evacuation time?
2. What are the effects of personal characteristics, namely age and gender, on building evacuation time?

## 2 Methods

### 2.1 Participants

In total 160 individuals participated, from which 7 participants were excluded in the analyses, due to computer problems with the administration of the surveys, not following the instructions given by the researchers or not correctly set evacuation conditions in the room where the experiments were conducted. Hence, the results discussed in this paper are based on 153 participants: 75 men (mean age 52.7, *SD* 16.7) and 78 women (mean age 48.9, *SD* 14.9). The participants were recruited via advertisements and news items in local newspapers in Arnhem and Velp (the Netherlands), via the distribution of flyers in several residential areas in Velp and via messages on Facebook sites of several local colleges, universities, scouting clubs and sporting clubs. Each participant received a financial reward of € 20. This study was approved by the Human Research Ethics Committee of Delft University of Technology.

### 2.2 Equipment: experience room and adjoining control room

**Fig. 1:**
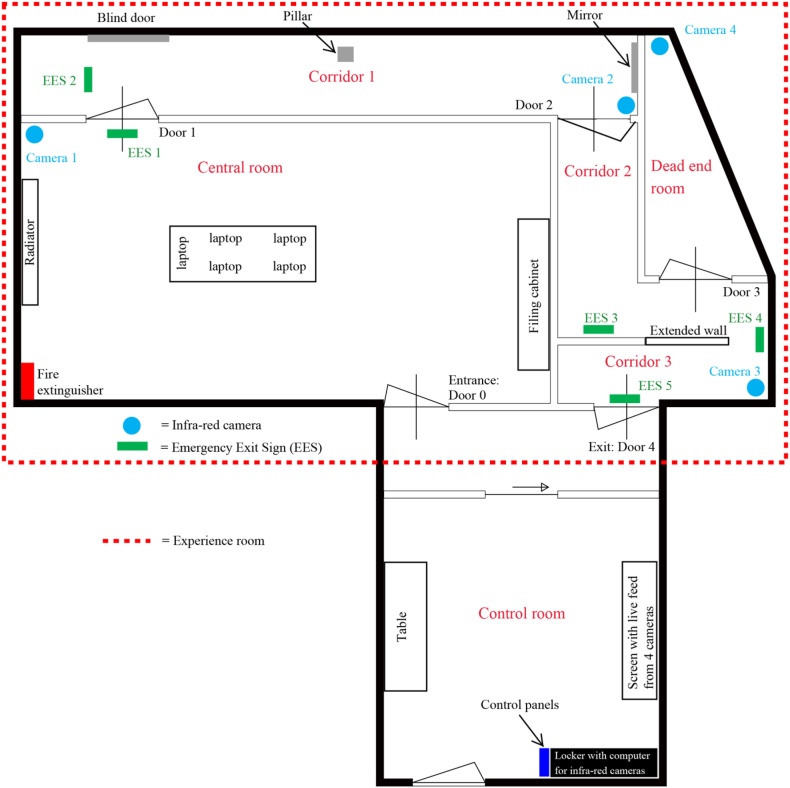
Floor plan of the experience room and adjoining control room.

The experiments were conducted in the experience room of a company specialized in emergency lighting in the Netherlands. The experience room was equipped with five green emergency exit signs, four infra-red video cameras (including a recording installation) and a sound installation system which contained the sound of a fire alarm. These instruments could be managed on several control panels, which were installed in the adjoining control room. See Fig. 1 for the floor plan of the experience room and the control room.

The experience room had four separated compartments without any windows. The first compartment is called the ‘central room’. This room contained a table with five laptops. There was also a filing cabinet on the right side and a fire extinguisher and radiator (cover) on the left side of the room. The second compartment, ‘corridor 1’, was a small corridor with a ‘blind door’ (i.e. this door could not be opened) at the left side and a mirror on the wall at the end of the corridor. There was a pillar in the middle of this corridor. The third compartment consisted of two corridors (‘corridor 2’ and ‘corridor 3’) which were partly separated by a wall. The fourth compartment was an empty room. The room could be entered through door 3, but it led nowhere. Therefore, this room is called the ‘dead end room’. Corridor 3 eventually led to the exit of the experience room, i.e. door 4.

In the control room, there were two control panels on which the fire alarm, different lighting levels and the emergency exit signs could be (de)activated. The activities of the participants in the experience room were monitored and recorded with four infra-red cameras (see Fig. 1 for the exact locations). The recordings were used to measure the evacuation time of the participants. This was the time between the moment the participant got up from his or her seat after completing a computer test and the moment the participant stepped through the last door opening (‘door 4’) at the end of corridor 3 (see Fig. 1).

Finally, at the start of the experiment the participants were welcomed and registered in the reception room (this room is not included in Fig. 1).

### 2.3 Experimental design

**Fig. 2:**
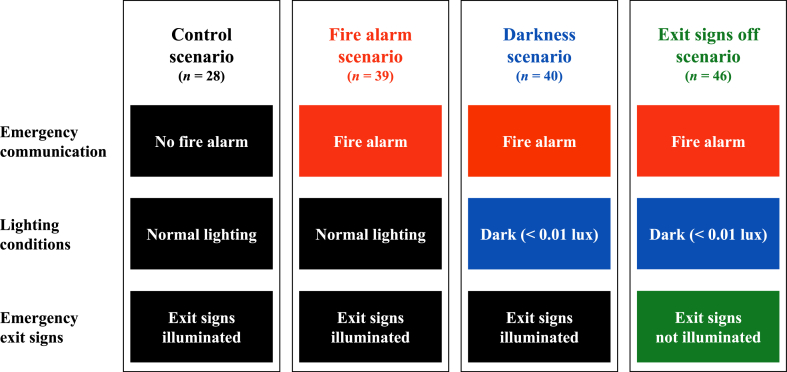
Set-up of the four evacuation scenarios.

In this study there was one control scenario and there were three experimental scenarios in which the following environmental factors were increasingly added to the control scenario: the sound of a fire alarm, darkness and not illuminated emergency exit signs (see Fig. 2).

In the control scenario all conditions in the experience room were normal: there was no fire alarm activated, the lighting was normal, and the five emergency exit signs were illuminated. In the first experimental scenario, called the ‘fire alarm scenario’, the fire alarm was activated, the lighting was normal, and the five emergency exit signs were illuminated. In the second experimental scenario, the ‘darkness scenario’, the fire alarm was activated, the lighting was set at a very low level (<0.01 lux), but it was not completely dark because of the light of the illuminated emergency exit signs. In the third experimental scenario, the ‘exit signs off scenario’, the fire alarm was activated, the lighting was set at a very low level (<0.01 lux), but also the five emergency exit signs were deactivated. The room was very dark and the emergency exit signs were not visible anymore. The online supplementary material contains photos of the lighting conditions in the experience room in the four evacuation scenarios (see Fig. 11 in Appendix A).

There were 28 participants in the control scenario, 39 participants in the fire alarm scenario, 40 participants in the darkness scenario and 46 participants in the exit signs off scenario.

In total, there were 38 experimental runs in which one to five individuals participated at the same time. Table 1 shows the distribution of the number of groups per group size and per scenario.

**Table 1:** Distribution of number of groups – per group size – in the four evacuation scenarios.

Group size	Evacuation scenario				Total
	Control	Fire alarm	Darkness	Exit signs off	
Group of 1	1	0	0	0	1
Group of 2	0	0	0	1	1
Group of 3	2	0	2	0	4
Group of 4	3	4	3	5	15
Group of 5	2	5	5	5	17
Total	8	9	10	11	38

### 2.4 Materials

**Fig. 3:**
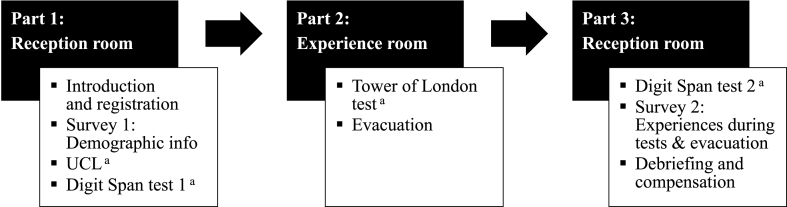
Overview of all used materials and the experimental procedure. *Note*. UCL = Utrecht Coping List. ^a^ Not analysed in this paper.

This study was part of a larger research study in which also (1) the effects of the three environmental factors on planning & problem-solving abilities and working memory functioning in evacuations were investigated as well as (2) the relationships between evacuation behaviour and planning & problem-solving abilities, working memory functioning and different coping styles. The results of these studies will be presented in separate manuscripts. An overview of the experimental procedure and all the administered psychological tests and surveys can be found in Fig. 3.

#### 2.4.1 Video recordings: evacuation time

The time it took participants to evacuate the experience room was recorded by four infra-red cameras. To be able to identify the participants during the analyses afterwards, the participants wore a reflecting band. In case of a maximum of five participants in an experiment, the first participant wore the band on the left upper arm, the second participant on the left forearm, the third on the right upper arm, the fourth on the right forearm and the last participant could then be identified by not wearing a band at all. The evacuation time measured was the time between the moment the participant got up from his or her seat after completion of the Tower of London test and the moment the participant stepped through the last door opening (‘door 4’) in the fourth compartment (see Fig. 1). This door led directly to the control room.

#### 2.4.2 Two surveys: personal characteristics and stress experiences

During the study two surveys were administered. The first survey was completed *before* the experiment in the experience room. It collected biographical information (gender, age) and the stress experience of the participants at that moment.

The second survey was completed *after* the experiment in the experience room and the participants reported their stress experiences for three other moments: (a) during the Tower of London test, (b) during evacuation and (c) the moment after the experiments. The results about the stress experiences of the participants *before* and *during* evacuation are discussed in Appendix B.

### 2.5 Procedure

The participants were welcomed in the reception room. There, they received a document including (1) information about the procedure of the experiment and that it could be possible that a fire alarm was activated and (2) an informed consent form. After reading this document, the participants were asked to sign the informed consent form. Then, they completed the first survey. Next, the participants were escorted by the researchers to the control room of the experience room. There, the mobile phones of the participants were collected or turned off and the reflecting bands were distributed (see section 2.4.1). Also, the researchers provided the participants with safety instructions. In case one of the participants did not feel comfortable in the experience room or did not want to participate anymore during the experiments they were instructed to put both arms in the air. In that case, the researchers in the control room would immediately stop the experiment. After giving these instructions, the participants were escorted to the central room of the experience room. Here, the participants received information about the Tower of London test. The participants were instructed that they, individually, were only allowed to leave the room and to find a way out of the experience room after they completed this test. They were also instructed not to use the door through which they entered the room. They had to find another way out of the experience room. Finally, the researchers told the participants that there was a present available in the control room for the person who successfully managed to evacuate the experience room first of his/her group. At the moment the researcher left the central room, another researcher started one of the experimental scenarios or did nothing in case of the control scenario. After the experiment in the experience room, the participants were immediately escorted to the reception room to complete the second survey.

## 3 Results

In the following sections the results of the experiments are presented per research question. The data is analysed with IBM SPSS Statistics (Version 29). The graphics were generated using DataGraph (Version 5.1.1) [42]. Between subjects ANOVAs were conducted for the effects of the three environmental factors and personal characteristics on building evacuation time. For the age-related analyses, participants were divided into six age categories (see section 3.2.1 for details about these age categories). Although the number of participants per evacuation scenario and/or age category differ, we chose ANOVAs for our analyses because normality should not be an issue of concern with sample sizes of 30+ [43,44]. For the post hoc analyses, Gabriel comparisons are given. These comparisons control the familywise error by correcting the level of significance for each test such that the overall Type I error rate across all comparisons remains at 0.05. The mentioned effect sizes are partial eta squared. As proposed by Cohen [45] the guidelines for interpreting (partial) eta squared effect sizes are: 0.01 = small effect, 0.06 = medium effect and 0.14 = large effect.

### 3.1 The effects of a fire alarm, darkness and not illuminated emergency exit signs on building evacuation time

**Fig. 4:**
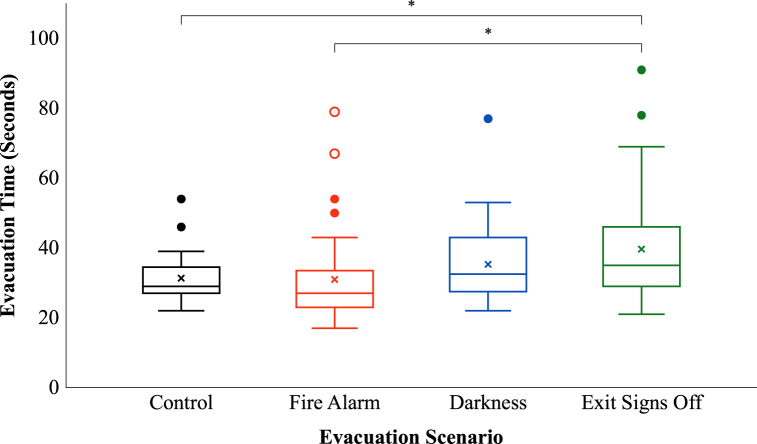
Evacuation time per evacuation scenario. *p < 0.05.

A one-way ANOVA revealed that the effect of the three environmental factors on evacuation time was significant at the *p* < 0.01 level, *F* (3, 149) = 4.17, *p* = 0.007, partial η^2^ = 0.08, a medium effect (see Fig. 4).

**Fig. 5:**
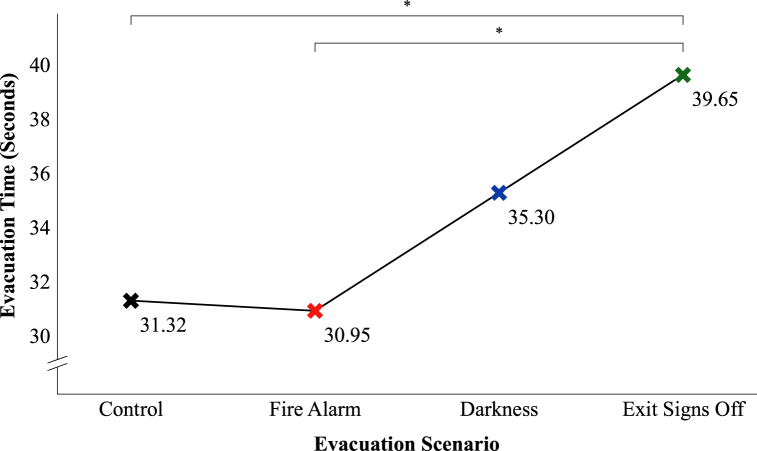
Zoomed-in trend line graph for evacuation time per evacuation scenario with mean values. *p < 0.05.

To have a better look at the differences between the mean values of the evacuation scenarios, in Fig. 5 a zoomed-in graph of the boxplots in Fig. 4 is shown. To see the upward trend, the mean values are connected with a trend line in Fig. 5.

**Fig. 6:**
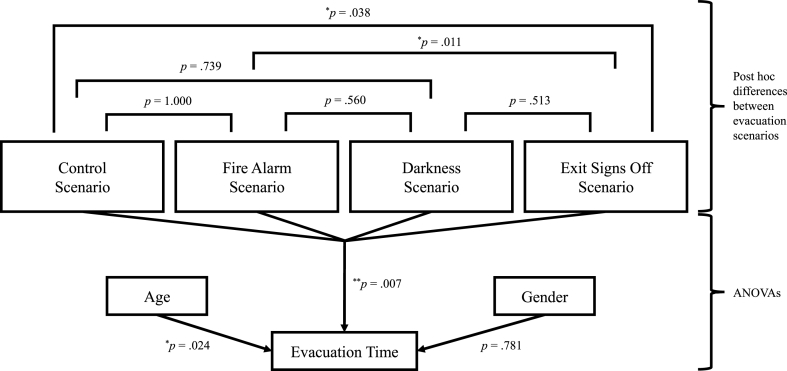
Effects of environmental factors, age and gender on evacuation time. *p < 0.05. **p < 0.01.

The post hoc tests revealed significant differences at the *p* < 0.05 level between the control and exit signs off scenario (*p* = 0.038) and between the fire alarm and exit signs off scenario (*p* = 0.011). Participants in the exit signs off scenario took on average 8 s more – an increase of 26.6% – to evacuate the experience room than participants in the control scenario and 8.5 s more – an increase of 28.1% – than the participants in the fire alarm scenario. This means that the combination of all the environmental factors (a fire alarm, darkness and not illuminated emergency exit signs) had a significant negative influence on the evacuation time, compared to no fire alarm, normal lighting conditions and illuminated emergency exit signs or only a fire alarm, normal lighting conditions and illuminated emergency exit signs. The other comparisons were not significantly different (control – fire alarm, *p* = 1.000, control – darkness, *p* = 0.739, fire alarm – darkness, *p* = 0.560, and darkness – exit signs off, *p* = 0.513). An overview of the results of these post hoc analyses is also presented in the upper part of Fig. 6.

### 3.2 The effects of personal characteristics on evacuation time

In this section the effects of age (section 3.2.1) and gender (section 3.2.2) on building evacuation time will be presented. In the two surveys, the participants were also asked about their personal stress experiences *before* and *during* the evacuation. In Appendix B of the online supplementary material the results of these analyses are reported.

#### 3.2.1 The effect of age on evacuation time

**Fig. 7:**
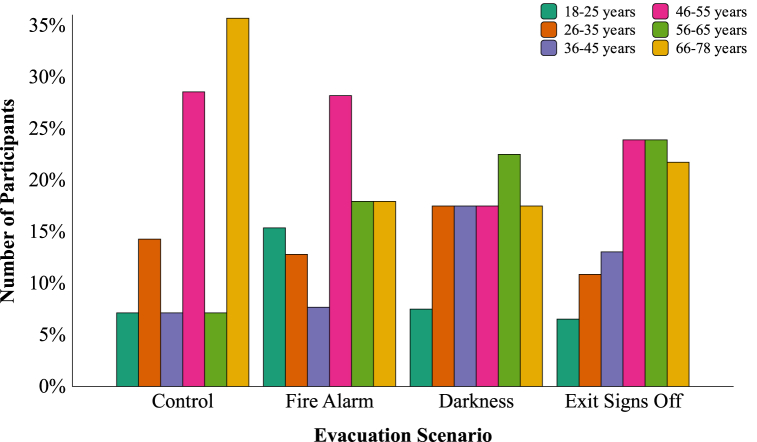
Distribution of participants per age category per evacuation scenario.

The participants were divided into six age categories according to their age. The ratio of the distribution of the different age categories over the four evacuation scenarios was not significantly different, χ^2^ (15) = 12.39, *p* = 0.649 (see Fig. 7 for the distribution).

**Fig. 8:**
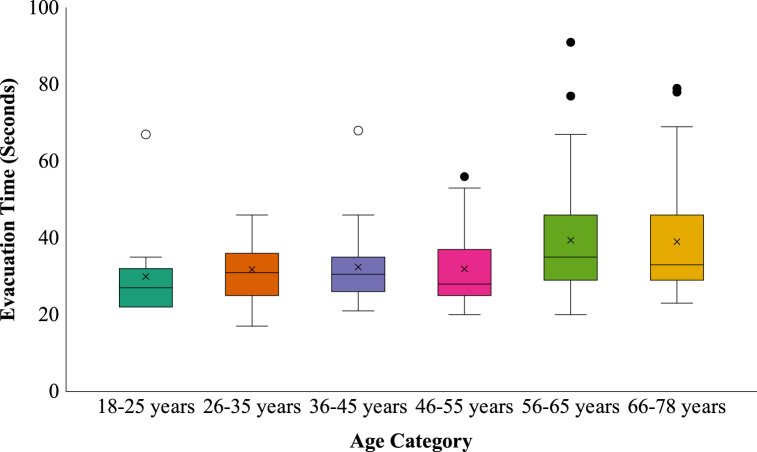
Evacuation time per age category.

There were not enough participants in the different age categories to compare these categories per evacuation scenario. However, it was possible to investigate if age category had an overall effect on evacuation time. A one-way ANOVA revealed that there was a significant effect of age on evacuation time at the *p* < 0.05 level, *F* (5, 147) = 2.67, *p* = 0.024, partial η^2^ = 0.08, a medium effect (see Fig. 8 and also Fig. 6, lower part).

**Fig. 9:**
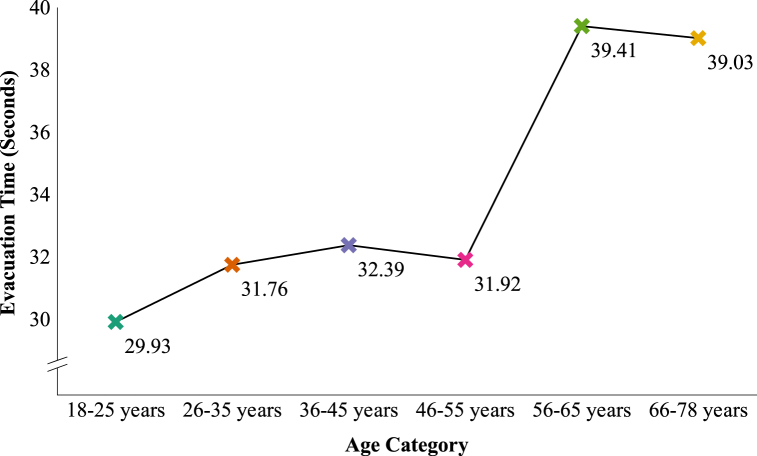
Zoomed-in trend line graph for evacuation time per age category with mean values.

To have a better look at the differences between the mean values of all age categories, in Fig. 9 a zoomed-in graph of the boxplots in Fig. 8 is shown. To see the upward trend, the mean values are connected with a trend line in Fig. 9.

There was a significant increase in evacuation time and this is especially the case for the ages of 56 years and older. The older participants needed much more time to evacuate the experience room than the younger participants. The difference between the slowest and the fastest age category, 56–65 years versus 18–25 years, is almost 9.5 s (an increase in evacuation time of 31.7%). The difference between the oldest participants (66–78 years) and the youngest participants (18–25 years) was comparable: more than 9 s (an increase in evacuation time of 30.4%). Although, the difference between the fastest and slowest age categories was 9.5 s, there were no significant post hoc tests.

#### 3.2.2 The effect of gender on evacuation time

**Fig. 10:**
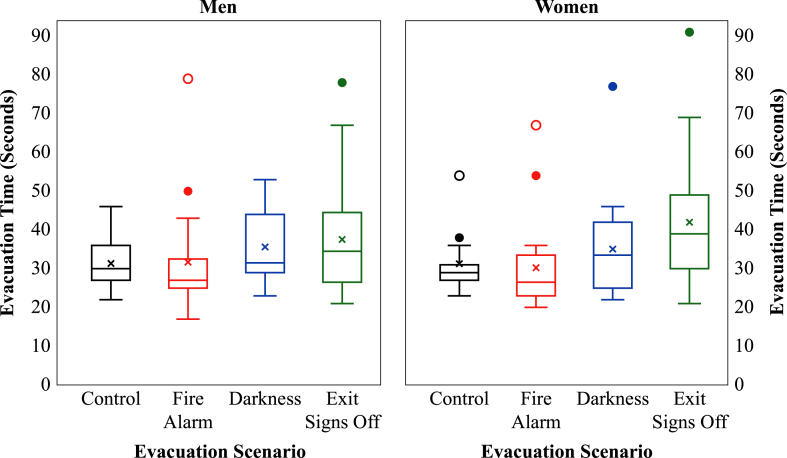
Differences between men and women on evacuation time per evacuation scenario.

The ratio of the distribution of men and women over the four evacuation scenarios was not significantly different, χ^2^ (3) = 0.45, *p* = 0.929. For gender it was possible to compare men and women per evacuation scenario. Therefore, a two-way ANOVA was conducted to explore the impact of the three environmental factors and gender on evacuation time. There was a significant main effect of the three environmental factors on evacuation time at the *p* < 0.01 level, *F* (3, 145) = 4.18, *p* = 0.007, partial η^2^ = 0.08, a medium effect. There was no main effect of gender on evacuation time, *F* (1, 145) = 0.08, *p* = 0.781 (see also Fig. 6, lower part). In the fire alarm and darkness scenarios women were on average a bit faster than men, but in the exit signs off scenario women were 4.5 s slower than men, meaning an increase in evacuation time of 11.7% (see Fig. 10). The interaction effect between the evacuation scenario and gender was not significant, *F* (3, 145) = 0.45, *p* = 0.715.

## 4 Discussion

In this experimental study the effects of three environmental factors on evacuation time were investigated. The chosen evacuation conditions were: (1) the use of a fire alarm in normal lighting conditions with illuminated emergency exit signs, (2) the use of a fire alarm combined with a dark environment and illuminated emergency exit signs and (3) the use of a fire alarm combined with a dark environment and not illuminated emergency exit signs. The main results indicate that the combination of a fire alarm, a dark environment and not illuminated emergency exit signs had a significant negative influence on the time it took participants to evacuate the experience room, compared to no fire alarm, normal lighting conditions and illuminated emergency exit signs or only a fire alarm, normal lighting conditions and illuminated emergency exit signs. Another important finding is that in this study age had a significant negative effect on evacuation time. For gender no significant effect was found.

### 4.1 Discussion results

#### 4.1.1 Evacuation time in different evacuation conditions

With the addition of environmental factors in this study, the evacuation time increased. Participants in the exit signs off scenario took on average 8 s more – an increase of 26.6% – to evacuate the room than participants in the control scenario. Also, compared to the fire alarm scenario, it took participants in the exit signs off scenario on average more than 8.5 s – an increase of 28.1%. These findings are in accordance with previous studies. Bode and Codling [29] conducted experiments in an interactive virtual environment and found that, when under pressure, participants were more likely to follow familiar routes and less likely to adapt their route choices, which resulted in longer evacuation times. In an immersive virtual museum experiment Lin et al. [46] found that in fire emergency conditions participants spent more time exiting the museum and travelled a longer distance than participants in normal conditions. Meng and Zhang [41] conducted a virtual hotel fire emergency experiment. In their treatment scenario a fire alarm and smoke in virtual reality was used combined with smoke in the real environment to provide olfactory and visual stimuli to the participants. They demonstrated that the participants in the treatment scenario travelled significant longer distances and experienced more stress than the control scenario. However, other studies show contradicting findings. Kobes et al. [10] performed evacuation experiments with three scenarios in a real hotel. There was a basic scenario with normal lighting levels, no smoke, and exit signs placed at ceiling level. The smoke scenario also had normal lighting levels, but now there was smoke visible, and the exit signs were at ceiling level. In the exit sign scenario the lighting levels were normal, there was smoke visible, but the exit signs were now at floor level. They found that the movement times in the smoke and exit sign scenarios were faster than in the basic scenario. In our study no smoke was used but in two scenarios the environment was dark. This might explain why in our study an increase in evacuation time was found. Daamen and Hoogendoorn [47] conducted experiments in which stress levels were raised by a slow-whoop signal or a combination of a slow-whoop signal and a stroboscope light. They found that a higher urgency led to higher evacuations speeds and a higher door capacity. Hence, evacuation was faster in the two stress scenarios compared to the no stress scenario. However, these experiments were conducted in a straightforward situation: the participants only had to walk through a door opening. In our study, participants had to find a way out of a room with four compartments. Haghani et al. [22] found in their experiments that the evacuation time of participants in the high stress scenario was shorter than of the participants in the low stress scenario. However, they only used a loud siren in the high stress scenario. Our experiment not only included a loud siren, but also darkness and not illuminated emergency exit signs.

#### 4.1.2 Evacuation time and age

Participants aged 56 years or older needed at least 9 s more – an increase of at least 30.4% – than the participants aged between 18 and 25 years to evacuate the room. This finding is in line with experiments conducted in a virtual environment by Zhiming et al. [30]. They found that the evacuation time tended to increase as the age of the participants increased. Also, in an underground evacuation study of Jeon et al. [48] it was found that the oldest age group (50–67 years) had the longest evacuation time in all the tests they performed compared to age groups 9–19 years and 20–49 years. In a study of Proulx and Pineau [49] in which evacuation times of a simulated fire emergency in two office buildings were compared with evacuation drills in apartment buildings, it was found that the mean evacuation speeds in the apartment buildings, compared to the office buildings, were reduced by the presence of children and elderly people. Because not a lot of studies could be found about the effects of age on evacuation or escape time specifically, also studies about travel/walking speed and wayfinding are included in this discussion. According to Boyce [28] older subjects tended to move over the escape route slightly more slowly than the younger group in both normal and dark illumination conditions. Experiments from Akizuki et al. [25] and Akizuki et al. [26] demonstrated that specifically in conditions with very low lighting levels (<3 lux) the travel speed of the younger participants is significantly higher than of the senior participants. In a research study of Head and Isom [50] regarding wayfinding and route learning skills in a virtual maze setup, it was found that age was associated with an increased distance travelled to locate landmarks in the maze. In the experiments of Bode and Codling [29] older participants seemed to have longer reaction times in a simulated evacuation experiment compared to younger participants. This means that it took older participants longer than younger participants to react in the virtual environment. It is not clear what the effect on their evacuation time specifically was.

#### 4.1.3 Evacuation time and gender

Gender did not have a significant effect on evacuation time. In the fire alarm and darkness scenarios women were a bit faster than men, but in the exit signs off scenario women were almost 4.5 s slower than men, meaning an increase in evacuation time of 11.7%. Studies about possible gender differences in evacuation times are scarce and therefore also studies about reaction time and travel/walking speed are included in this discussion. In the experiments of Bode and Codling [29] women appeared to have longer reaction times compared to men. This means that it took women longer than men to react in the virtual environment. It is not clear what the effect on their evacuation time specifically was. Zhiming et al. [30] investigated the effect of gender on evacuation time and their results revealed that it took women significantly longer than men to evacuate. Fuji et al. [27] investigated walking speeds in smoke-filled corridors and they showed that the walking speeds of men were higher than of women, except for total darkness or in a dark condition with only a lit emergency exit sign. However, it is not clear what the effect on evacuation time specifically was.

### 4.2 Strengths, limitations and future research

An advantage of an experimental set-up is that the environment can be controlled and that the exhibited behaviour can be monitored and observed closely. A strength of this study was the set-up of the experiments. The evacuation conditions increased per scenario and there were four scenarios, a control scenario and three experimental scenarios. In evacuation experiments usually fewer scenarios are compared [9,41,51]. Another strength of this research is that the effect of the combination of the three environmental factors on building evacuation time has been quantified. More research is needed in this respect but in the future calculations for expected evacuation times could take these kind of factors into account. Furthermore, a diverse group of participants was used. The participants came from the community and the age range was from 18 to 78 years. In a lot of evacuation experiments students are the test subjects [22,34,46,52] but in our study a diverse group of people from the community was involved. Although the distribution of the participants in the six age categories over the four evacuation scenarios was not significantly different, we recommend for future research to have more balanced age categories.

A disadvantage of an experimental study is that the participants are aware that it is an experiment. Therefore, these kind of studies should also be performed in more ‘real life’ circumstances, such as during unannounced fire drills. Another recommendation for future research is to include irritating smoke in test scenarios. In most fires people perish because of smoke inhalation [16,53,54] and the combination of a dark environment and irritating smoke would even more resemble a real fire emergency.

### 4.3 Practical and theoretical implications

A practical recommendation for, for example, building managers and safety managers is that it should be considered in evacuation plans that evacuations in darkness can take considerably longer than evacuations in normal lighting conditions. Of course, this should also be tested in the buildings and locations itself, but in our study a dark environment and not illuminated emergency signs accounted for an increase in evacuation time of more than 25%.

Another practical recommendation for building managers is to consider the population in their buildings. An older population in buildings could mean that evacuations can take longer than anticipated in existing evacuation plans. In our study, the increase in evacuation time for participants of 56 years and older was more than 30% compared to participants of 18–25 years.

A theoretical and methodological recommendation for future research about evacuation behaviour is to also investigate the effects of and relationships with personal characteristics such as gender and age. Also psychological characteristics such as stress experiences or coping styles in stressful conditions should be included in this kind of research. In previous studies these human factors are rarely investigated in evacuation experiments.

## 5 Conclusions

Our findings suggest that especially the combination of a fire alarm, a dark environment and not illuminated emergency exit signs in an emergency leads to an increase in evacuation time compared to no fire alarm, normal lighting conditions and illuminated emergency exit signs or only a fire alarm, normal lighting conditions and illuminated emergency exit signs. Also, age appears to have a negative effect on evacuation time. The effect of gender is not so clear, but our findings warrant more research about this. These findings also highlight the importance of including personal and psychological factors in evacuation research in addition to technical data such as evacuation time, reaction time, walking speed, etc. A multidisciplinary approach is recommended for this kind of research.

## Funding statement

The research presented in this paper has been sponsored by the Dutch Foundation of Scientific Research NWO-MaGW.

## Ethics statement

This study was approved by the Human Research Ethics Committee of Delft University of Technology.

## CRediT authorship contribution statement

**Erica Kinkel:** Writing – review & editing, Writing – original draft, Visualization, Methodology, Investigation, Formal analysis, Conceptualization. **C. Natalie van der Wal:** Writing – review & editing, Writing – original draft, Visualization, Formal analysis. **Serge P. Hoogendoorn:** Writing – review & editing, Funding acquisition.

## Declaration of competing interest

The authors declare that they have no known competing financial interests or personal relationships that could have appeared to influence the work reported in this paper.
