# Supplementary material for: The effects of three environmental factors on building evacuation time
Source: Heliyon. 2024 Feb 24;10(5):e27128. doi: 10.1016/j.heliyon.2024.e27128 (PMC10943335; doi:10.1016/j.heliyon.2024.e27128)
Supplement: Multimedia component 1 [file mmc1.pdf]

Online supplementary material – Appendix A

1. Central room	2. Corridor 1	3. Corridor 1	4. Corridor 2	5. Corridor 2	6. Dead end room	7. Corridor 3
**Control and fire alarm scenario**						
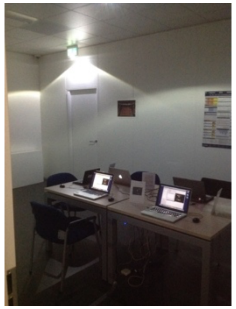	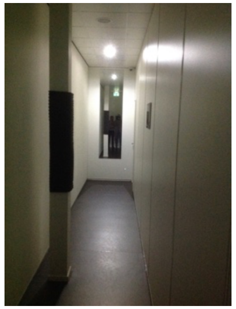	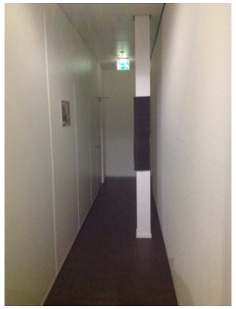	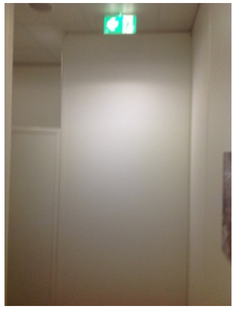	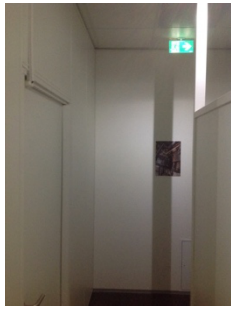	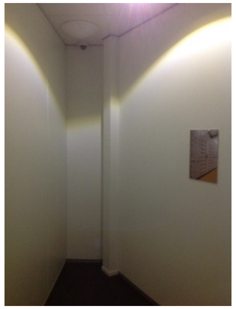	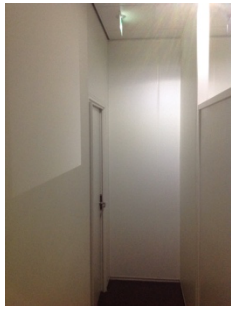
**Darkness scenario**						
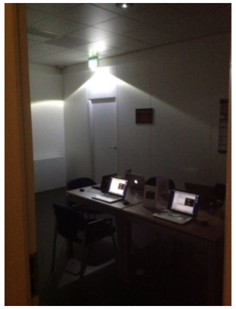	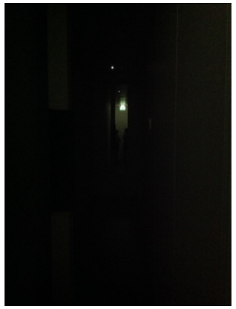	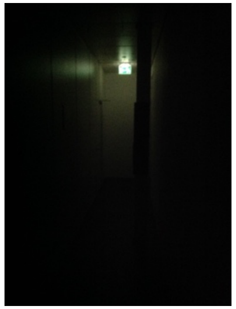	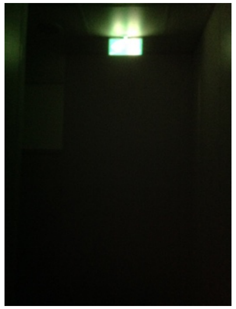	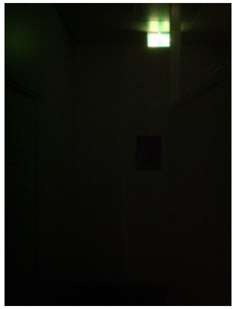	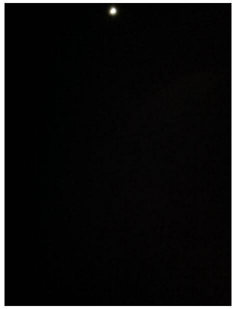	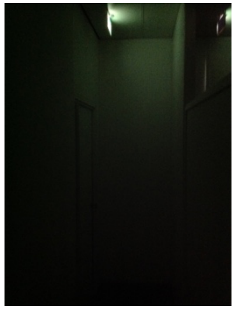
**Exit signs off scenario**						
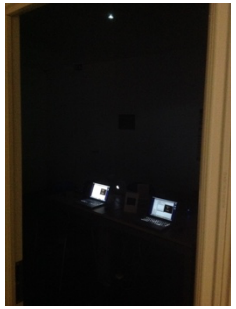	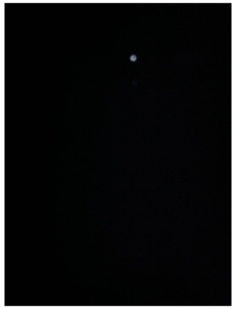	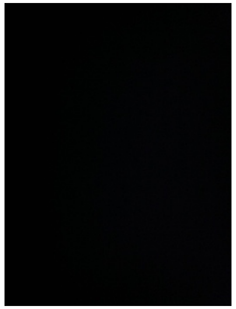	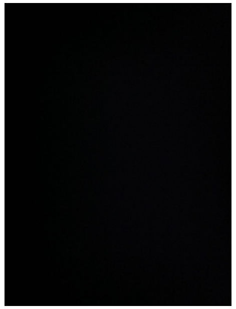	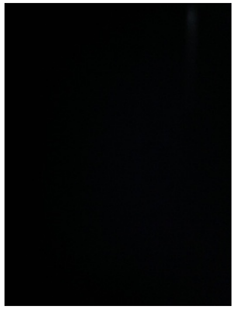	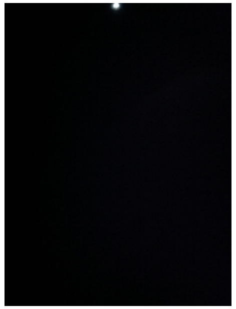	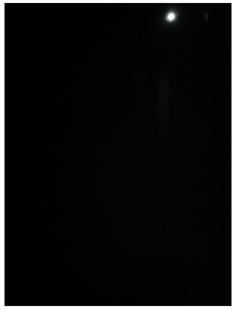

Fig. 11. Photos of the lighting conditions in the experience room in the control, fire alarm, darkness and exit signs off scenarios.

Online supplementary material – Appendix B

In the two administered surveys, the participants were, amongst others, inquired about their stress experiences *before* and *during* the evacuation. The participants reported their subjective experiences of stress on a scale from 0 (*no stress*) to 10 (*very much stress*).

The differences between the stress experiences *before* and *during* evacuation are analysed per evacuation scenario and per gender with mixed between-within subjects ANOVAs.

Stress experiences before and during evacuation per evacuation scenario

A mixed ANOVA was conducted to assess the impact of the evacuation scenario on the subjective stress experiences of the participants across two moments in time, namely *before* and *during* evacuation.

There was no significant main effect of moment in time on the stress experiences, $F(1, 149) = 3.23$, $p = .071$, indicating that the participants did not differ significantly in their stress experiences *before* and *during* evacuation. The main effect comparing the four evacuation scenarios on the stress experiences *before* and *during* the evacuation was not significant either, $F(3, 149) = 0.44$, $p = .728$. Neither was there an interaction effect between moment in time and evacuation scenario, $F(3, 149) = 0.67$, $p = .570$.

Stress experiences before and during evacuation per gender and evacuation scenario

A mixed ANOVA was conducted to assess the impact of gender on the subjective stress experiences of the participants across two moments in time, namely *before* and *during* evacuation.

There was a significant main effect of moment in time on the stress experiences at the $p < .05$ level, $F(1, 151) = 4.08$, $p = .045$, partial $\eta^2 = 0.03$, a small effect. This indicates that men and women differ significantly in their stress experiences *before* and *during* evacuation. The main effect comparing men and women on the stress experiences *before* and *during* the evacuation was significant at the $p < .001$ level, $F(1, 151) = 16.37$, partial $\eta^2 = 0.10$, a medium to large effect. The mean difference between the stress experiences *before* and *after* evacuation for women was significantly higher than for men. There was no interaction effect between moment in time and gender, $F(1, 151) = 0.74$, $p = .391$.

To further investigate the difference between men and women, mixed ANOVAs for the impact of the evacuation scenario were conducted for men and women separately. For men, there was a significant main effect of moment in time on their stress experiences at the $p < .05$ level, $F(1, 71) = 4.43$, $p = .039$, partial $\eta^2 = 0.06$, a medium effect. Men experienced significant more stress *during* evacuation than *before* the evacuation (see Fig. 12). The main effect comparing the four evacuation scenarios on the stress experiences of men *before* and *during* the evacuation was not significant, $F(3, 71) = 1.02$, $p = .390$. Neither was there an interaction effect for men between moment in time and evacuation scenario, $F(3, 71) = 1.12$, $p = .348$. For women, there was no significant main effect of moment in time on their stress experiences, $F(1, 74) = 0.46$, $p = .501$. Although in almost all evacuation scenarios women did experience more stress *during* evacuation than *before* the evacuation, this was not the case in the exit signs off scenario (see Fig. 12). There was also no main effect comparing the four evacuation scenarios on the stress experiences of women *before* and *during* the evacuation, $F(3, 74) = 1.27$, $p = .290$.

Neither was there an interaction effect for women between moment in time and evacuation scenario, $F(3, 74) = 0.41$, $p = .746$.

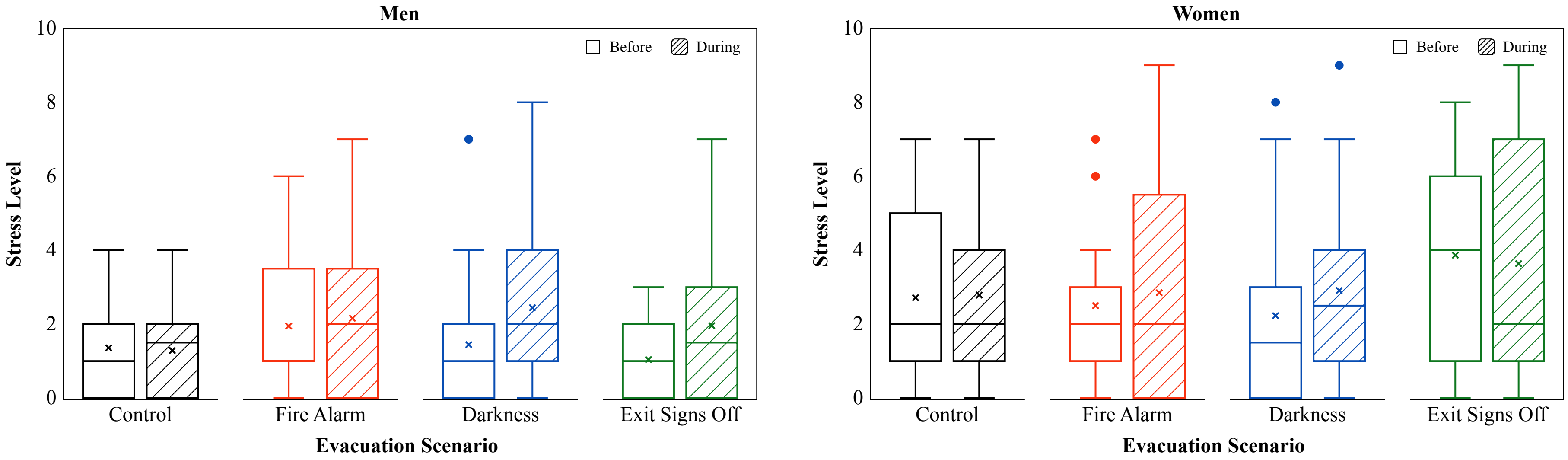

Fig. 12. Stress experiences men and women before and during evacuation per evacuation scenario.
